# Subungual Melanoma: A Single Institution Experience

**DOI:** 10.3390/medsci9030057

**Published:** 2021-09-15

**Authors:** Christopher J. LaRocca, Lily Lai, Rebecca A. Nelson, Badri Modi, Brooke Crawford

**Affiliations:** 1 Department of Surgery, University of Minnesota, Minneapolis, MN 55455, USA; 2 City of Hope National Medical Center, Department of Surgery, Duarte, CA 91010, USA; llai@coh.org (L.L.); bamodi@coh.org (B.M.); 3 City of Hope National Medical Center, Department of Computational and Quantitative Medicine, Duarte, CA 91010, USA; rnelson@coh.org; 4 UCLA Medical Center, Department of Orthopedic Surgery, Santa Monica, CA 90404, USA

**Keywords:** subungual, melanoma, acral lentiginous, nail, skin cancer

## Abstract

Despite the changing paradigms of melanoma treatment in recent years, there remains a relative paucity of data regarding subungual melanoma in the literature. From 2002–2018, 25 patients with subungual melanoma were surgically treated at our facility. A retrospective chart review was conducted to collect relevant demographic, clinical, pathologic, and outcomes data. The median age at diagnosis was 69 years. Most patients (60%) were male, and the melanoma lesion was most often located on the foot (68%). Acral-lentiginous was the most common histologic subtype (59%), and the median Breslow thickness was 3.4 mm. Fifteen patients (63%) underwent a sentinel lymph node biopsy as part of their surgical resection, and four of these patients (27%) had metastatic disease in the lymph nodes. In total, 10 patients underwent lymph node dissection of the involved basin. The median follow up was 21 months in this patient population. Age, gender, tumor location, ulceration, and lesion histology were not significantly associated with recurrence free survival (RFS). Increasing Breslow thickness was found to be significantly associated with shorter RFS (HR: 1.07, CI: 1.03–1.55). In total, 13 patients developed a disease recurrence, and RFS rates were 66% at 1 year and 40% at 3 years. Additionally, 91 and 37% of patients were alive at one year and three years, respectively. Subungual melanomas are rare lesions that often have a more advanced stage at diagnosis, which contributes to the poor prognosis of these cutaneous malignancies.

## 1. Introduction

The melanoma treatment landscape has undergone widespread changes in recent years with the emergence of immune checkpoint blockade and therapies targeting the BRAF pathway [1,2], the Food and Drug Administration (FDA) approval of the first oncolytic virus (Talimogene laherperepvec [T-VEC]) [3], and the move away from completion lymph node dissections secondary to the results of the second Multicenter Selective Lymphadenectomy Trial (MSLT-2) [4]. However, despite these shifts in treatment paradigms, there remains a relative paucity of data regarding subungual melanomas in the literature.

Subungual melanomas arise from the nail apparatus [5] and are rare tumors with an annual incidence of approximately 0.1 per 100,000 individuals [6], which accounts for up to 3% of all cutaneous melanomas [7]. Interestingly, there are differences in the rates of subungual melanoma between different racial and ethnic groups where they account for approximately 1–2% of all cutaneous melanomas in non-Hispanic Caucasians, but they can account for upwards of 20% in Asian and African American populations [6,8,9,10]. Both whole genome and next generation sequencing have revealed that acral melanomas harbor mutational signatures that are unique from other cutaneous melanomas (often a lower tumor mutational burden), demonstrating less reliance on the BRAF pathway and ultraviolet radiation as sources of carcinogenesis [11,12]. Additionally, hair salon UV lamps and recurrent trauma have been suggested as having a potential role in the pathogenesis of subungual melanoma [13,14].

Subungual melanomas present unique clinical challenges, in part due to diagnostic difficulties resulting in delays in diagnosis or misdiagnosis [15,16]. Due to these challenges, many subungual melanomas are identified at later stages and consequently have worse prognoses and survival outcomes compared to cutaneous melanomas subtypes [10,17]. Five year overall survival rates have been reported to range from 15–59%, depending on the series [17,18].

In this report, we describe our institutional series of 25 subungual melanoma patients, including the demographic makeup, clinicopathologic characteristics, and treatment factors associated with survival.

## 2. Materials and Methods

### 2.1. Patients

From 2002–2018, 25 patients with subungual melanoma were surgically treated at City of Hope National Medical Center, a National Cancer Institute (NCI) designated Comprehensive Cancer Center. After obtaining approval from our Institutional Review Board (IRB), we conducted a retrospective chart review to collect relevant demographic, clinicopathologic, treatment-related, and outcome data. Recurrence free survival (RFS) was calculated as the time from the date of diagnosis to the first evidence of disease recurrence. If the patient did not experience a recurrence, they were censored at their last tumor follow-up date. Overall survival was calculated from their date of diagnosis to their date of death. Patients alive at last contact were censored at their last contact date.

### 2.2. Statistical Analysis

Descriptive statistics were used to characterize the patient population. Univariate Cox proportional hazards models were used to determine the association between demographic and clinical risk factors with recurrence-free and overall survival. Survival was further characterized using Kaplan–Meier plots. All analyses were performed using SAS version 9.4 (SAS Institute Inc., Cary, NC, USA).

## 3. Results

### 3.1. Demographic and Histopathologic

There were 25 patients included in this retrospective study with a median age at diagnosis of 69 years (Table 1). The majority of patients were male (60%), and the melanoma lesion was located on the foot (68%) more often than on the hand. Of those patients with histology reports available for review (17 of 25), acral-lentiginous was the most common histologic subtype (59%), followed by nodular (18%) and superficial spreading (6%). Additionally, 18% of patients had in situ disease. For those patients with Breslow thickness available for review (18 of 25), the median thickness was 3.4 mm, with 50% of the patients having intermediate thickness melanomas (2–4 mm) and 30% having a thick melanoma (>4 mm). When recorded in the pathology reports, 76% of lesions were found to be ulcerated and 69% had an elevated mitotic index (>1 per mm^2^). No patient had AJCC Stage IV disease, and the majority of patients (52%) were found to have Stage III disease after their initial surgical resection.

### 3.2. Regional and Systemic Management

Fifteen patients (63%) underwent a sentinel lymph node biopsy as part of their surgical resection. Of the remaining 10 patients, three had in situ disease, six went directly to a completion lymph node dissection (due to the presence of clinically positive/palpable lymph nodes upon initial presentation), and one patient’s sentinel node status was unknown. Four patients (27%) who underwent a sentinel lymph node biopsy had evidence of metastatic disease upon pathologic review and subsequently underwent a completion lymph node dissection of the involved basin. Eight patients (33%) went on to receive adjuvant therapy in the form chemotherapy (dacarbazine-based), immunotherapy (interferon alpha, ipilimumab, or pembrolizumab), radiation therapy to the regional nodal basin, or a melanoma vaccine trial (Supplementary Table S1).

### 3.3. Recurrence Free Survival

**Figure 1:**
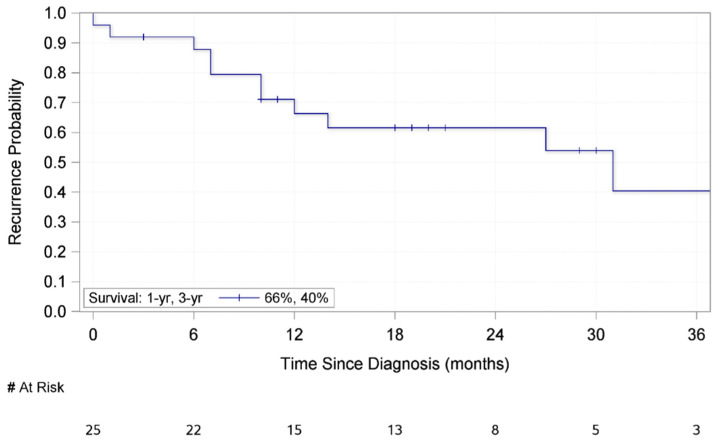
Kaplan–Meier Curve for Recurrence Free Survival of 25 patients who Underwent Surgical Resection of a Subungual Melanoma.

The median follow up was 21 months (interquartile range (IQR): 18–29 months) in this patient population. Table 2 outlines the association between demographic and clinicopathologic factors and recurrence-free survival (RFS). Age, gender, tumor location (hand or foot), tumor laterality, ulceration, and lesion histology were not significantly associated with RFS. Increasing Breslow thickness was found to be significantly associated with shorter RFS (HR: 1.27, CI: 1.03–1.55). Additionally, patients who underwent a completion lymph node dissection either at time of presentation with clinically matted/palpable nodes or with a positive sentinel lymph node biopsy demonstrated a marginally significant association with shorter RFS (HR: 2.87, CI: 0.83–9.89). The mean RFS was 33.6 months. Additionally, the median RFS of all patients in this study was 31 months (IQR: 10–41 months) and RFS rates were 66% at 1 year and 40% at 3 years (Figure 1). In the study population, four patients recurred with in-transit disease, four developed a regional nodal basin recurrence, and six developed distant metastases as their initial recurrence (Supplementary Table S1).

### 3.4. Overall Survival

**Figure 2:**
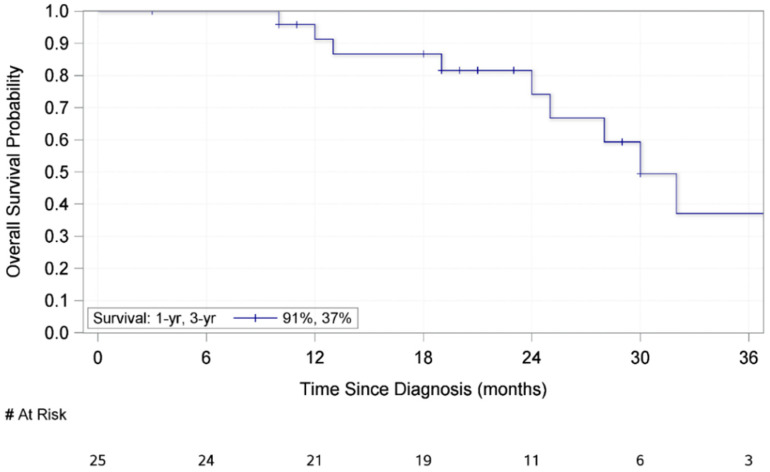
Kaplan–Meier Curve for Overall Survival of 25 Patients who Underwent Surgical Resection of a Subungual Melanoma.

Table 3 outlines the association between demographic and clinicopathologic factors and overall survival. No variable had a statistically significant association with overall survival; however, a marginally significant association was found between increasing Breslow thickness and shorter overall survival (HR 1.17, CI: 1.00–1.37). For these patients, the mean overall survival was 48.4 months. Furthermore, the median overall survival was 30 months (IQR: 24–81 months) (Figure 2) with 91% and 37% of patients alive at one year and three years, respectively, after initial resection. In total, there were 11 deaths in this patient population, and nine of these were attributed to melanoma disease progression.

## 4. Discussion

When compared to non-nail apparatus cutaneous melanoma, subungual melanoma represents a rare disease subtype with a relative paucity of clinical data. We present our institutional experience on subungual melanoma to add to the collective literature and confirm key trends in patient presentation and survival.

The incidence of melanoma has been increasing in recent years, and the vast majority of melanoma patients have thin melanomas that are less than 1 mm in depth [19,20]. This is in contrast to patients with subungual melanomas who typically present at later stages and have even been found to have worse survival outcomes when staged-matched and compared to non-acral cutaneous melanomas [21]. For instance, the median Breslow thickness in our patient population was 3.4 mm, which is consistent with previously published series of subungual melanoma [6,7]. In addition, we found the most common histologic subtype of subungual melanoma to be acral lentiginous (59%), and this is comparable to other institutional series [6,22,23]. Furthermore, the rate of positive sentinel lymph node biopsies increases with the thickness of the primary lesions [24]. Our data demonstrates that 26.7% of patients in our study had a positive sentinel lymph node biopsy, which is within the range reported by other series [9,22,25]. In total, 40% of patients in our study had metastases in regional nodal basins on final pathologic analysis.

The overall survival rate in our study was 37% at three years. This is somewhat less than other reports in the literature which have demonstrated near 60% survival rate at five years [7,18]. However, similar to other studies, our data demonstrates a marginally significant association between shorter survival and regional nodal metastases [6]. Additionally, increasing Breslow thickness was associated with shorter RFS (*p* < 0.05).

All the patients in our series (except for one individual with in situ disease who had a wide local excision) underwent an amputation of the affected digit. Historically, amputation has been the treatment of choice with much debate centering on the level of amputation necessary to maximize survival. Some reports have demonstrated that the level of amputation does not matter so long as that the surgical margins are negative [7,18]. Additionally, there has been a push to further minimize the extent of resection when possible and to avoid amputation in favor of wide local excision [26]. In one of the largest series of conservative surgical approach, Moehrle et al. did not find any significant decrease in overall survival for patients who underwent local excision (compared to amputation); however, the local excision group did have a lower mean lesion thickness [27].

One of the limitations of this study is its retrospective nature with certain information not available in patients’ chart, despite an exhaustive review of print and electronic medical records. The patients were referred and treated at a tertiary cancer center and may not reflect the general population. In addition, the limited number of patients included in this study (partially a result of the rarity of the disease) and non-uniform distribution of certain variables limited the statistical power of the study.

## 5. Conclusions

In conclusion, our study results add to the body of literature and support findings previously published on subungual melanoma. Patients are often diagnosed at later disease stages, and this finding likely contributes to the unfavorable prognosis of subungual melanoma, which is particularly poor in those patients with thick primary lesions and lymph node metastases. Given the unique molecular characteristics and tumor microenvironment of subungual melanoma (when compared to other cutaneous melanomas), future studies will need to identify the adjuvant therapy regimens that will be most efficacious for patients with advanced disease.

## Figures and Tables

**Table 1:** Baseline Patient Characteristics.

		All
		N = 25
		N (%)
Age at Diagnosis	Median (IQR^+^)	69 (58–76)
Sex	Male	15 (60)
	Female	10 (40)
Histologic Subtype	Acral-lentiginous	10 (59)
	Melanoma in situ	3 (18)
	Nodular	3 (18)
	Superficial Spreading	1 (6)
	Unknown/NR	8
Breslow Thickness (mm)	Median (IQR^+^)	3.4 (2.3–4.3)
Breslow Thickness (mm)	<2	4 (20)
	2–4	10 (50)
	>4	6 (30)
	In situ	3
	Unknown/NR	2
AJCC Stage	0	3 (13)
	I	2 (9)
	II	6 (26)
	III	12 (52)
	IV	0
	Unknown/NR	2
Laterality	Right	10 (40)
	Left	15 (60)
Hand/Foot	Hand	8 (32)
	Foot	17 (68)
Ulceration	Present	13 (76)
	Absent	4 (24)
	Unknown/NR	8
PNI	Yes	1 (4.0)
	No	8 (32.0)
	Unknown/NR	16 (64.0)
LVI	Yes	2 (8.0)
	No	8 (32.0)
	Unknown/NR	15 (60.0)
Mitotic Index (>1 per mm^2^)	Present	9
	Absent	4
	Unknown/NR	12
SLNB Performed	Yes	15 (63)
	No	9 (38)
	Unknown/NR	1
Lymph Node Dissection	Yes	10 (42)
	No	14 (58)
	Unknown/NR	1
Received Adjuvant Therapy	Yes	8 (33)
	No	16 (67)
Disease Recurrence	Yes	13 (52)
	No	12 (48)

IQR: Interquartile Range, NR: Not Recorded, PNI: Perineural Invasion, LVI: Lymphovascular Invasion, SLNB: Sentinel Lymph Node Biopsy.

**Table 2:** Univariate Cox Proportional Hazards on Recurrence Free Survival.

		All N = 25 N (%)	Hazard Ratio (95% CI)	*p*-Value
Age at Diagnosis	Median (IQR^+^)	69 (58–76)	1.01 (0.97–1.05)	0.5492
Sex	Male	15 (60)	-reference-	-
	Female	10 (40)	1.29 (0.34–4.90)	0.7119
Breslow Thickness (mm)	Median (IQR^+^)	3.4 (2.5–4.5)	1.27 (1.03–1.55)	0.0228
Laterality	Right	10 (40)	-reference-	-
	Left	15 (60)	1.40 (0.40–4.93)	0.6027
Hand/Foot	Hand	8 (32)	-reference-	-
	Foot	17 (68)	0.64 (0.18–2.28)	0.4935
AJCC Stage	0-II	11 (44)	-reference-	-
	III	12 (48)	3.89 (0.83–18.32)	0.0859
	Unknown/NR	2 (8)	9.80 (0.82–117.06)	0.0713
Ulceration	Yes	13 (52)	-reference-	-
	No	4 (16)	2.47 (0.45–13.53)	0.2965
	Unknown/NR	8 (32)	1.49 (0.35–6.28)	0.5844
Histology	Acral-lentiginous	10 (40)	-reference-	-
	Melanoma in situ	3 (12)	UND	UND
	Nodular	3 (12)	2.68 (0.44–16.39)	0.2872
	Superficial Spreading	1 (4)	4.42 (0.44–44.48)	0.2074
	Unknown/NR	8 (32)	2.34 (0.52–10.61)	0.2707
PNI	No	8 (32)	-reference-	-
	Yes	1 (4)	1.24 (0.11–13.41)	0.8601
	Unknown/NR	16 (64)	0.94 (0.23–3.92)	0.9351
LVI	No	8 (32)	-reference-	-
	Yes	2 (8)	1.47 (0.23–9.50)	0.6842
	Unknown/NR	15 (60)	0.86 (0.20–3.75)	0.8446
SLNB	No	9 (36)	-reference-	-
	Yes	15 (60)	0.57 (0.17–1.91)	0.3649
	Unknown/NR	1 (4)	UND	UND
CLND	No	14 (56)	-reference-	-
	Yes	10 (40)	2.87 (0.83–9.89)	0.0950
	Unknown/NR	1 (4)	UND	UND
Adjuvant Therapy	No	16 (64)	-reference-	-
	Yes	8 (32)	0.97 (0.26–3.68)	0.9687
	Unknown/NR	1 (4)	UND	UND

IQR: Interquartile Range, NR: Not Recorded, PNI: Perineural Invasion, LVI: Lymphovascular Invasion, SLNB: Sentinel Lymph Node Biopsy, CLND: Completion Lymph Node Dissection.

**Table 3:** Univariate Cox Proportional Hazards on Overall Survival.

		All N = 25 N (%)	Hazard Ratio (95% CI)	*p*-Value
Age at Diagnosis	Median (IQR^+^)	69 (58–76)	1.03 (0.98–1.09)	0.2694
Sex	Male	15 (60)	-reference-	-
	Female	10 (40)	1.27 (0.30–5.43)	0.7443
Breslow Thickness (mm)	Median (IQR^+^)	3.4 (2.3–4.3)	1.17 (1.00–1.37)	0.0576
Laterality	Right	10 (40)	-reference-	-
	Left	15 (60)	1.70 (0.42–6.94)	0.4610
Hand/Foot	Hand	8 (32)	-reference-	-
	Foot	17 (68)	0.70 (0.17–2.83)	0.6161
AJCC Stage	0-II	11 (44)	-reference-	-
	III	12 (48)	5.52 (0.68–45.12)	0.1108
	Unknown/NR	2 (8)	29.45 (2.24–386.65)	0.0100
Ulceration	Yes	13 (52)	-reference-	-
	No	4 (16)	2.95 (0.27–31.93)	0.3726
	Unknown/NR	8 (32)	2.05 (0.51–8.24)	0.3116
Histology	Acral-lentiginous	10 (40)	-reference-	-
	Melanoma in situ	3 (12)	UND	UND
	Nodular	3 (12)	5.63 (0.49–65.08)	0.1662
	Superficial Spreading	1 (4)	10.00 (0.59–169.87)	0.1112
	Unknown/NR	8 (32)	5.71 (0.67–48.94)	0.1120
PNI	No	8 (32)	-reference-	-
	Yes	1 (4)	2.10 (0.12–37.76)	0.6152
	Unknown/NR	16 (64)	2.06 (0.24–17.63)	0.5107
LVI	No	8 (32)	-reference-	-
	Yes	2 (8)	2.43 (0.20–29.69)	0.4861
	Unknown/NR	15 (60)	1.98 (0.23–17.30)	0.5357
SLNB	No	9 (36)	-reference-	-
	Yes	15 (60)	0.40 (0.11–1.49)	0.1713
	Unknown/NR	1 (4)	2.43 (0.25–24.10)	0.4468
CLND	No	14 (56)	-reference-	-
	Yes	10 (40)	2.53 (0.62–10.30)	0.1955
	Unknown/NR	1 (4)	7.05 (0.64–78.24)	0.1116
Adjuvant Therapy	No	16 (64)	-reference-	-
	Yes	8 (32)	0.90 (0.21–3.96)	0.8932
	Unknown/NR	1 (4)	3.97 (0.41–38.75)	0.2349

IQR: Interquartile Range, NR: Not Recorded, PNI: Perineural Invasion, LVI: Lymphovascular Invasion, SLNB: Sentinel Lymph Node Biopsy, CLND: Completion Lymph Node Dissection.

## Data Availability

Data is contained within the article and supplementary material.

## Supplementary Materials

The following are available online at https://www.mdpi.com/article/10.3390/medsci9030057/s1, Table S1: Supplementary Table-Patient Treatment Factors.

## Sample Availability

Samples of the compounds are not available from the authors.
