# Supplementary material for: Subungual Melanoma: A Single Institution Experience
Source: Med Sci (Basel). 2021 Sep 15;9(3):57. doi: 10.3390/medsci9030057 (PMC8482220; doi:10.3390/medsci9030057)
Supplement: Supplementary file 1 [file medsci-09-00057-s001.zip › medsci-1337366-supplementary.pdf]

Patient	Age	Gender	Laterality	Location	AJCC Stage	Breslow Thickness (mm)	Ulceration	Mitoses (per mm^2)	Histology	Year of Surgery	SLNB	Size of Largest SLNB Metastasis (mm)	Lymph Node Dissection	Adjuvant Therapy	Site of Initial Recurrence	RFS (months)	Status
1	70	F	Left	Finger	0				MIS	2017	N/A		N/A				NED
2	18	F	Right	Finger	0				MIS	2017	N/A		N/A				NED
3	86	M	Right	Toe	0				MIS	2016	N/A		N/A				NED
4	52	M	Right	Finger	IA	0.8	NR	NR	AL	2006	Negative		N/A				NED
5	61	M	Right	Toe	IB	1.2	No	0	AL	2018	Negative		N/A				NED
6	77	F	Right	Finger	IIA	3.8	No	1	NR	2008	Negative		N/A		In-transit	7	DOD
7	74	M	Left	Toe	IIB	3.2	Yes	7	AL	2004	Negative		N/A				NED
8	61	M	Left	Finger	IIB	5.5	No	8	AL	2017	Negative		N/A		In-transit	14	NED
9	89	M	Right	Toe	IIB	3.7	Yes	5	AL	2016	Negative		N/A				NED
10	56	F	Left	Toe	IIB	2.9	Yes	6	AL	2015	Negative		N/A				NED
11	74	M	Right	Toe	IIC	4.5	Yes	NR	NR	2002	Negative		N/A	Clinical Trial - Vaccine	In-transit, Nodal	73	Dead (Unknown Cause)
12	75	M	Left	Finger	IIIA	0.4	No	<1	AL	2016	Positive	< 1	N/A	None			NED
13	58	F	Left	Toe	IIIC	4.1	Yes	3	AL	2016	Positive	1.7	Positive	Clinical Trial - Pembrolizumab			NED
14	74	M	Left	Toe	IIIC	2.1	Yes	0	SS	2012	Positive	1.0	Negative	Patient Declined	Distant Metastasis	12	DOD
15	20	F	Left	Toe	IIIC	3.0	Yes	NR	Nodular	2005	Positive	NR	Negative	Interferon alpha			NED
16	54	M	Right	Toe	IIIC	4.0	Yes	6	AL	2014	Positive	3.0	N/A	Ipilimumab	Distant Metastasis	31	DOD
17	66	M	Left	Finger	IIIC	4.0	NR	NR	Nodular	2001	N/A		Positive	Clinical Trial - Dacarbazine, Cisplati	Distant Metastasis	27	DOD

														n, Vinblastine, IL-2, Interferon			
18	61	F	Right	Toe	IIIC	15	Yes	12	AL	2017	Positive	13	Positive	NR	Nodal	10	AWD
19	51	M	Left	Toe	IIID	30	yes	NR	Nodular	2004	N/A		Positive	Interferon alpha	Distant Metastasis	6	DOD
20	59	M	Left	Toe	IIIC	2.7	yes	NR	NR	2004	N/A		Positive	Dacarbazine	Distant Metastasis	41	DOD
21	77	M	Left	Finger	III	NR	NR	NR	NR	2013	N/A		Positive	Radiotherapy	In-transit	1	DOD
22	84	F	Left	Toe	IIID	6.0	Yes	>1	NR	2013	N/A		Positive	Patient Declined			NED
23	81	F	Left	Toe	III	NR	NR	NR	NR	2012	N/A		Positive	Patient Declined	Nodal	7	DOD
24	69	F	Right	Toe	NR	1.5	Yes	4	NR	2012	Negative		N/A		Distant Metastasis	10	DOD
25	76	M	Left	Toe	NR	2.5	NR	NR	NR	2008	NR		NR		Nodal	NR	Dead (Unknown Cause)

MIS: melanoma in situ, AL: acral lentiginous, SS: superficial spreading, NED: no evidence of disease, DOD: died of disease, AWD: alive with disease, SLNB: sentinel lymph node biopsy, NR: not recorded, N/A: not applicable.
